# Hole-Free Nested Array with Three Sub-ULAs for Direction of Arrival Estimation

**DOI:** 10.3390/s23115214

**Published:** 2023-05-30

**Authors:** Yule Zhang, Guoping Hu, Hao Zhou, Juan Bai, Chenghong Zhan, Shuhan Guo

**Affiliations:** 1 Graduate College, Air Force Engineering University, Xi’an 710051, China; yule_zhang0921@163.com (Y.Z.); chenghong_zhan@163.com (C.Z.); guoshuhan0304@163.com (S.G.); 2 Air and Missile Defense College, Air Force Engineering University, Xi’an 710051, China; 17792611529@126.com (H.Z.); b_juan@163.com (J.B.)

**Keywords:** direction of arrival estimation, sparse array, hole-free nested array with three sub-ULAs, difference co-array, degrees of freedom

## Abstract

Sparse arrays are of deep concern due to their ability to identify more sources than the number of sensors, among which the hole-free difference co-array (DCA) with large degrees of freedom (DOFs) is a topic worth discussing. In this paper, we propose a novel hole-free nested array with three sub-uniform line arrays (NA-TS). The one-dimensional (1D) and two-dimensional (2D) representations demonstrate the detailed configuration of NA-TS, which indicates that both nested array (NA) and improved nested array (INA) are special cases of NA-TS. We subsequently derive the closed-form expressions for the optimal configuration and the available number of DOFs, concluding that the DOFs of NA-TS is a function of the number of sensors and the number of the third sub-ULA. The NA-TS possesses more DOFs than several previously proposed hole-free nested arrays. Finally, the superior direction of arrival (DOA) estimation performance based on the NA-TS is supported by numerical examples.

## 1. Introduction

Direction of arrival (DOA) estimation, as an important means to obtain source angles, has made significant progress [1,2,3,4,5]. However, the uniform linear arrays (ULAs) [6] and uniform planar arrays [7,8,9] are typically utilized in conventional sensor direction-finding systems to avoid spatial aliasing, whose decreased degrees of freedom (DOFs) and huge hardware overhead no longer meet the practical needs and development of the current orientation systems.

Sparse arrays [10,11,12,13] have been favored by many scholars and engineers for their excellent properties such as enhanced DOFs [10], reduced mutual coupling [11] and low redundancy [12]. In the content of the difference co-array [14], sparse arrays can detect more sources than physical sensors, providing a new perspective for DOA estimation.

The best-known and fundamental sparse geometry is the nested array (NA) [10], which acquires hole-free lags in the yielding difference co-array, thereby promoting the application and development of the subspace-based techniques [10,15,16,17]. Afterwards, extensive efforts have been devoted to enhancing the attainable number of uniform DOFs (uDOFs). An improved nested array (INA) was proposed in [18], which adjusts the inter-sensor spacing between the inner and outer sub-ULAs and adds an additional sensor at the end to provide enhanced uDOFs. In [19], an extension of NA (EoNA) with larger array aperture and more uDOFs was developed by shifting the *N*_2_-1 sensors at the end of NA backward to the unit underlying grid. Enhanced Nested Array (ENA) was constructed in [20] by arranging a dense ULA and a sparse ULA on both sides of a single sensor. Moreover, super nested array (SNA) [21], augmented nested array (ANA) [22], generalized nested array (GNA) [23] and enhanced generalized nested array (EGNA) [24] have been designed from alleviating the mutual coupling; SNA and GNA cannot improve the DOFs, and holes exist in both ANA, GNA and EGNA.

Another popular sparse geometry is the coprime array (CPA) [11], which suppresses the mutual coupling due to its larger inter-sensor spacing but provides a smaller number of uDOFs as compared with NA. This array and its variants [25,26,27,28,29,30] have also been widely studied, but they inevitably encounter discontinuous lags in the generating difference co-array. Although various approaches, such as compressed sensing (CS) [31], virtual array interpolation [32,33] and sensor array motions [34,35], have been proposed to solve this problem, CS and virtual array interpolation bear a huge computational burden, and sensor array motions require a quasi-stationarity environment where the source locations are considered invariant over array motion of half wavelength or multiples half wavelength. Therefore, the subspace-based techniques such as [10,15,16,17] are still the most direct and efficient estimation algorithms.

In this paper, a novel nested array with three sub-ULAs (NA-TS) is proposed for DOA estimation. NA-TS can offer a large number of uDOFs and its hole-free feature makes it very compatible with subspace-based algorithms. We derive the closed-form expression for NA-TS and compare the advantages over existing hole-free nested arrays.

The paper outline is as follows: The complete process of sparse array signal processing is introduced in Section 2. In Section 3, the configuration of NA-TS is defined, and its properties are investigated. Simulation results are presented in Section 4. Finally, the conclusions are drawn in Section 5.

## 2. Sparse Array Signal Processing

In this section, we focus on the complete process of sparse array signal processing, which mainly includes the signal model, difference co-array and DOA estimation methods. Meanwhile, we also introduce some related terminologies to help readers understand the paper.

### 2.1. Signal Model

Let us consider a sparse array with N sensors fixed at the locations S×λ/2, where S and λ denote the normalized location set and source wavelength, respectively. Location set S is an integer set

(1) $$S=\left\{l_{n}\left|n=0,1,\cdots ,N-1\right.\right\}$$

For the sparse array, the steering vector for a certain direction θ is expressed as $a\left(\theta \right)={\left[e^{-j\left(2\pi /\lambda \;\right)l_{0}d_{0}\sin\theta },e^{-j\left(2\pi /\lambda \;\right)l_{1}d_{0}\sin\theta },\cdots ,e^{-j\left(2\pi /\lambda \;\right)l_{N-1}d_{0}\sin\theta }\right]}^{T}$, where $j=\sqrt{-1}$ represents the imaginary unit. $d_{0}$ represents the unit inter-sensor spacing. ${\left[\cdot \right]}^{T}$ represents the transpose operator.

Assume that K far-field narrowband and uncorrelated sources impinge on the sparse array S from directions $\Theta =\left\{\theta_{k}\left|k=1,2,\cdots ,K\right.\right\}$. To be specific, there are sources $\left\{s_{k}\left(t\right)\left|k=1,2,\cdots ,K\right.\right\}$ with powers $\left\{\sigma_{k}^{2}\left|k=1,2,\cdots ,K\right.\right\}$, where t=1,2,⋯,L, and L is the number of sampling snapshots. Then, the array output, at snapshot t, is modeled as

(2) $$x\left(t\right)=\sum_{k=1}^{K}s_{k}\left(t\right)a\left(\theta_{k}\right)+n\left(t\right)=As\left(t\right)+n\left(t\right)$$

where A is the array manifold matrix with the *k*th column being $\left\{a\left(\theta_{k}\right)\left|k=1,2,\cdots ,K\right.\right\}$. $s\left(t\right)={\left[s_{1}\left(t\right),s_{2}\left(t\right),\cdots ,s_{K}\left(t\right)\right]}^{T}$ is the source vector. $n\left(t\right)={\left[n_{1}\left(t\right),n_{2}\left(t\right),\cdots ,n_{N}\left(t\right)\right]}^{T}$ is the additive white noise vector following the complex Gaussian distribution $CN\left(0,\sigma_{n}^{2}I_{N}\right)$, which is independent of the sources. $\sigma_{n}^{2}$ represents the noise power, and $I_{N}$ represents the N×N-dimensional identity matrix.

### 2.2. Difference Co-Array

Under the above assumptions, the covariance matrix of $x\left(t\right)$ can be calculated as

(3) $$R=E\left[x\left(t\right)x^{H}\left(t\right)\right]=AR_{s}A^{H}+\sigma_{n}^{2}I_{N}$$

where $R_{s}=E\left[s\left(t\right)s^{H}\left(t\right)\right]=\mathrm{diag}\left[\sigma_{1}^{2},\sigma_{2}^{2},\cdots ,\sigma_{K}^{2}\right]$ is the source covariance matrix. The term $\sigma_{n}^{2}I_{N}$ is the noise covariance matrix. $E\left[\cdot \right]$ represents the expectation operator. ${\left[\cdot \right]}^{H}$ represents the Hermitian transpose operator. $\mathrm{diag}\left[\cdot \right]$ forms a diagonal matrix from the entries.

Note that in practice, (3) can be approximated by the average of multiple sampling snapshots

(4) $$\hat{R}\approx \frac{1}{L}\sum_{t=1}^{L}x\left(t\right)x^{H}\left(t\right)$$

Then, following the Khatri–Rao processing [14], we vectorize (3) to yield the following model

(5) $$r=\mathrm{vec}\left(R\right)=\left(A^{*}\circ A\right)p+\sigma_{n}^{2}\mathrm{vec}\left(I_{N}\right)=Bp+\sigma_{n}^{2}i$$

where $p={\left[\sigma_{1}^{2},\sigma_{2}^{2},\cdots ,\sigma_{K}^{2}\right]}^{T}$. $i=\mathrm{vec}\left(I_{N}\right)={\left[e_{1}^{T},e_{2}^{T},\cdots ,e_{N}^{T}\right]}^{T}$ with $\left\{e_{i}\left|i=1,2,\cdots ,N\right.\right\}$ being a column vector of 1 in the *i*th row and 0 in the rest. The symbol ∘ represents the Khatri–Rao product. ${\left[\cdot \right]}^{*}$ represents the conjugate operator. $\mathrm{vec}\left(\cdot \right)$ represents the vectorization operator.

Comparing (2) and (5), r can be viewed as an output of a virtual array whose manifold matrix is expressed as $A^{*}\circ A$. The virtual array is the well-known difference co-array whose sensor locations are given by the difference set

(6) $$D=\left\{l_{m}-l_{n}\left|m,n=0,1,\cdots ,N-1\right.\right\}$$

Next, we will define several useful terminologies regarding the difference co-array.

**Definition 1 (DOFs):**  *For a given sparse array* S, *the DOFs is the cardinality of its difference co-array* D, i.e. , *DOFs* = $\left|D\right|$.

**Definition 2 (uDOFs):**  *For a sparse array* S, *let* U *represent the largest consecutive segment around zero in* D, *then the cardinality of*  U *is termed uDOFs, i.e., uDOFs* =$\left|U\right|$.

**Definition 3 (Hole):**  *The smallest consecutive lags containing* D *is defined as* $V≜\left\{m\left|\min\left(D\right)\leq m\leq \max\left(D\right)\right.\right\}$. *Thus, an integer h is considered as a hole in the difference co-array if*  h∈V *but* h∉D.

**Definition 4 (Restricted Array):**  *A restricted array refers to an array without holes in its difference co-array. In other words, for a restricted array, we have* D=U=V.

It is obvious that $\left|V\right|\geq \left|D\right|\geq \left|U\right|$, where the equal sign is taken if and only if the difference co-array is hole-free.

Now, let us proceed to consider the single-snapshot model. Note that duplicate elements and holes are allowed in D, we need to use a $\left|U\right|\times N^{2}$-dimensional selection matrix J to update (5)

(7) $$y=Jr=B_{1}p+\sigma_{n}^{2}e$$

where J is a binary matrix with only a 1 in each row, whose position is determined by the index of the selected element among D. $B_{1}$ denotes a $\left|U\right|\times K$-dimensional array manifold matrix of a virtual ULA. The term $e={\left[0_{1\times \left(\left|U\right|-1\right)/2},1,0_{1\times \left(\left|U\right|-1\right)/2}\right]}^{T}$.

### 2.3. DOA Estimation

Assuming ${\left[y\right]}_{i}$ represents the $i+\left(\left|U\right|+1\right)/2$ th component of vector y, we construct the Toeplitz matrix as follows:

(8) $$T=\left[\begin{array}{cccc} {\left[y\right]}_{0} & {\left[y\right]}_{-1} & \cdots & {\left[y\right]}_{-\left(\left|U\right|-1\right)/2}\\ {\left[y\right]}_{1} & {\left[y\right]}_{0} & \cdots & {\left[y\right]}_{-\left(\left|U\right|-1\right)/2+1}\\ \vdots & \vdots & \ddots & \vdots \\ {\left[y\right]}_{\left(\left|U\right|-1\right)/2} & {\left[y\right]}_{\left(\left|U\right|-1\right)/2-1} & \cdots & {\left[y\right]}_{0} \end{array}\right]$$

Subsequently, applying the MUSIC [2] or ESPRIT [3] algorithm on T can resolve up to $\left(\left|U\right|-1\right)/2$ DOAs.

## 3. Nested Array with Three Sub-ULAs (NA-TS)

As we all know, nested array provides more DOFs than the number of sensors by systematically nesting two sub-ULAs with different inter-sensor spacing, thereby obtaining accurate estimation performance. To acquire more DOFs, a novel nested array with three sub-ULAs, named as NA-TS, is proposed in this section.

### 3.1. Configuration

**Figure 1:**
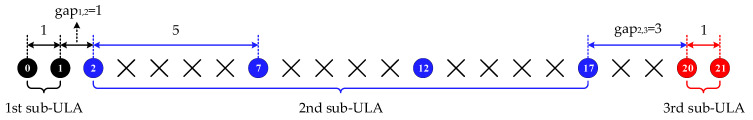
Nested array with three sub-ULAs (NA-TS), where we assume *N*_1_ = 2, *N*_2_ = 4 and *N*_3_ = 2.

It is well known that the INA is constructed by two ULAs and an additional sensor, and the resulting high DOFs is more conducive to DOA estimation. Inspired by this, we generalize the additional sensor at the end into an $N_{3}$-sensor uniform linear array with an interspacing of $d_{0}$. To obtain a hole-free difference co-array, we still preserve the distance between adjacent sub-arrays. Here, we use $gap_{i,j}$ to represent the distance between the first sensor in the *j*th sub-ULA and the last sensor in the *i*th sub-ULA. Thus, we have $gap_{1,2}=d_{0}$ and $gap_{2,3}=\left(N_{1}+1\right)d_{0}$, respectively. In this case, the distance between the last sensor in the 3rd sub-ULA and the last sensor in the 2nd sub-ULA is $\left(N_{1}+N_{3}\right)d_{0}$. Therefore, to further enhance the DOFs, we can set the interspacing of the 2nd sub-ULA to $\left(N_{1}+1+N_{3}\right)d_{0}$. Then, we obtain the NA-TS shown in Figure 1, whose normalized sensor locations can be defined by the following set S, i.e.,

(9) $$S=S_{1}\cup S_{2}\cup S_{3}$$

where

(10) $$\left\{\begin{array}{l} S_{1}=\left\{s_{1}\left|s_{1}=0,1,\cdots ,N_{1}-1\right.\right\}\\ S_{2}=\left\{N_{1}+\left(N_{1}+1+N_{3}\right)s_{2}\left|s_{2}=0,1,\cdots ,N_{2}-1\right.\right\}\\ S_{3}=\left\{N_{1}+\left(N_{1}+1+N_{3}\right)\left(N_{2}-1\right)+\left(N_{1}+1\right)+s_{3}\left|s_{3}=0,1,\cdots ,N_{3}-1\right.\right\} \end{array}\right.$$

**Remark 1.**  *According to (10) and (11), we can observe that, in case of N_3_ = 0, the proposed array configuration degenerates into NA. If we set N_3_ = 1, the INA configuration can be constructed* *. Thus, both NA and INA can be interpreted as special cases of the NA-TS.*

**Figure 2:**
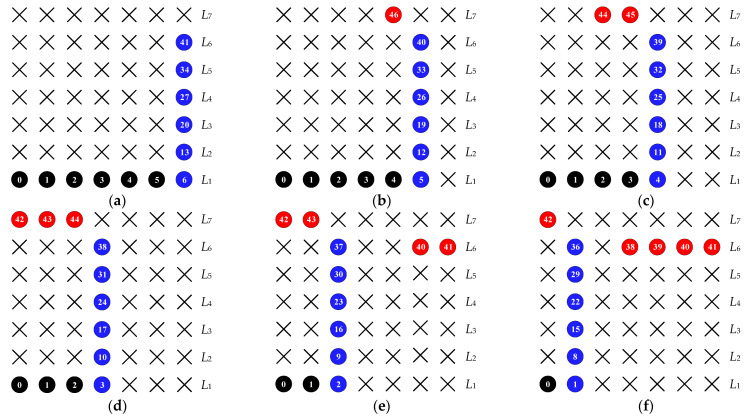
2D representations of (**a**) NA, (**b**) NA-TS (*N*_3_ = 1), (**c**) NA-TS (*N*_3_ = 2), (**d**) NA-TS (*N*_3_ = 3), (**e**) NA-TS (*N*_3_ = 4), and (**f**) NA-TS (*N*_3_ = 5), where *N* = 12. The black, blue and red circles with numbers denote the 1st sub-ULA, 2nd sub-ULA and 3rd sub-ULA sensor locations, respectively, while crosses indicate empty space.

**Remark 2.**  *To further understand the NA and the proposed NA-TS, Figure 2 shows the two-dimensional (2D) representations (defined in [21]) of the two sparse arrays, where* $L_{i}$ *represents the ith layer defined as the positions from* $\left(i-1\right)\left(N_{1}+1+N_{3}\right)$ *to* $i\left(N_{1}+1+N_{3}\right)-1$. *It is appreciated from the 2D representations that NA-TS is generated by rearranging the elements of the NA in the* $\left(N_{1}+1+N_{3}\right)\times \left(N_{2}+1\right)=7\times 7$ -*layer plane, thus leading to improved DOFs and array aperture*.

### 3.2. Properties

**Definition 5.**  *The difference co-array of the proposed NA-TS is defined as*

(11) $$D=D^{+}\cup D^{-}$$

*where* $D^{+}=\left\{s-\tilde{s}\left|s,\tilde{s}\in S,s\geq \tilde{s}\right.\right\}$ *and* $D^{-}=\left\{\tilde{s}-s\left|s,\tilde{s}\in S,s\geq \tilde{s}\right.\right\}$.

Based on the Definition 5, the properties of NA-TS are derived as the following proposition.

**Proposition 1.**  *In case of* $N_{1}\geq 1,\;N_{2}\geq 2,\;N_{3}\geq 1$*, the proposed NA-TS is a restricted array, and *$2\left(N_{1}N_{2}+N_{2}N_{3}+N_{1}+N_{2}\right)-1$ *DOFs can be obtained*.

Proof. See Appendix A. □

**Proposition 2.**  *Given an*$N=N_{1}+N_{2}+N_{3}$*-sensor NA-TS, the closed-form expression for the number of DOFs is a function of* N *and*  $N_{3}$*, i.e.,* $\left|D\right|=N^{2}/2+2N-2N_{3}-1$ *in case of*  N *is even, and* $\left|D\right|=N^{2}/2+2N-2N_{3}-3/2$ *in case of* N *is odd*.

Proof. See Appendix B. □

### 3.3. Comparisons

The optimal DOFs and corresponding solutions for NA-TS and other restricted nested arrays (including NA [10], EoNA [19], ENA [20]) are summarized in Table 1. We can see that, like other configurations, the NA-TS has a simple closed-form expression for DOFs, while possesses more DOFs and effective virtual aperture. Particularly, the maximum DOFs of NA-TS is obtained in case of *N*_3_ = 1.

## 4. Simulation Results

In this section, extensive numerical examples are provided to evaluate the superiority of NA-TS, where CPA [11], NA [10], EoNA [19] and ENA [20] are selected as contrasts.

### 4.1. Degrees of Freedom

**Figure 3:**
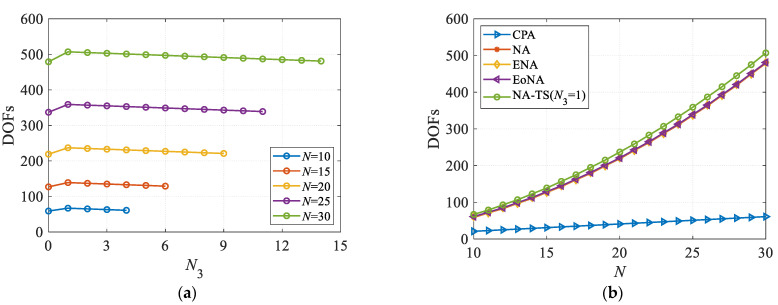
Comparisons of DOFs. (**a**) DOFs versus *N*_3_ for NA-TS; (**b**) DOFs versus *N* for different configurations.

In the first numerical example, we evaluate the DOFs property for different configurations. In Figure 3a, the DOFs of NA-TS are plotted as a function of *N*_3_ for different numbers of sensors. Note that NA-TS degenerates into NA when *N*_3_ = 0. We can draw the conclusion that for NA-TS with any number of sensors, the DOFs are greater than those in NA in case of *N*_3_ ≥ 1 as well that as the maximum DOFs are available if *N*_3_ = 1. Furthermore, the curves of the DOFs with the number of sensors for different configurations are plotted in Figure 3b. It can be found that CPA results in the least number of DOFs due to the holes, while both NA and its variants can obtain more DOFs, and the proposed NA-TS has more DOFs than that of other nested configurations. This numerical example verifies the validity of the DOFs property for the different nested configurations summarized in Table 1.

### 4.2. MUSIC Spectra

**Figure 4:**
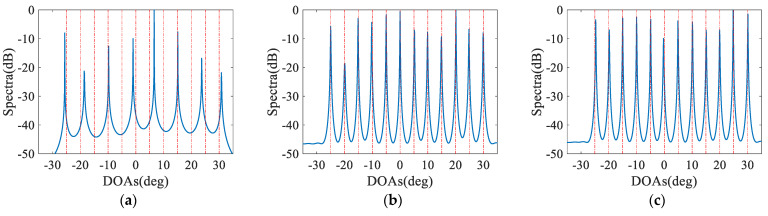

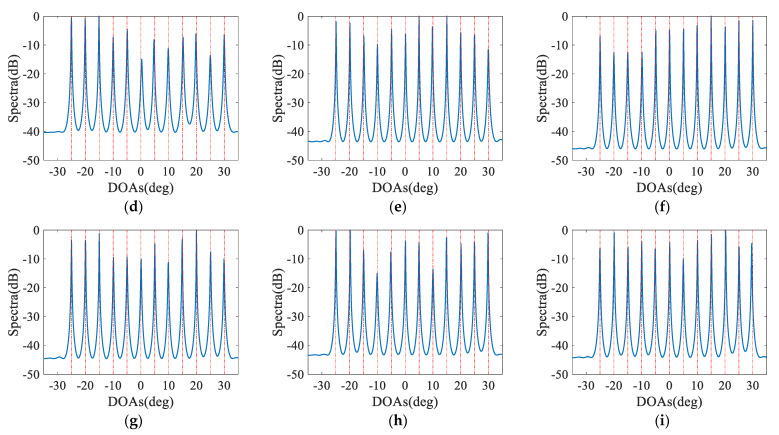
Comparison of spatial spectra, where *N* = 12, *K* = 12, SNR = 0 dB and *L* = 500. The blue solid lines and the red dashed lines represent the estimation results and true DOAs, respectively. (**a**) CPA; (**b**) NA; (**c**) ENA; (**d**) EoNA; (**e**) NA-TS (*N*_3_ = 1); (**f**) NA-TS (*N*_3_ = 2); (**g**) NA-TS (*N*_3_ = 3); (**h**) NA-TS (*N*_3_ = 4); (**i**) NA-TS (*N*_3_ = 5).

In the second numerical example, we plot the MUSIC spectra for different configurations in Figure 4, which considered here to consist of 12 sensors. Since the maximum estimable number of sources for the CPA is 12, we estimate 12 sources distributed from −25° to 30° with a step of 5°, where the signal-to-noise ratio (SNR) is 0 dB and the number of snapshots equals 500. It is observed that CPA fails to identify the 12 sources as it performs the case of the maximum number of estimable sources, while all nested configurations can accurately identify the 12 sources, which is attributed to the more DOFs and the effective virtual aperture.

### 4.3. Resolution Ability

**Figure 5:**
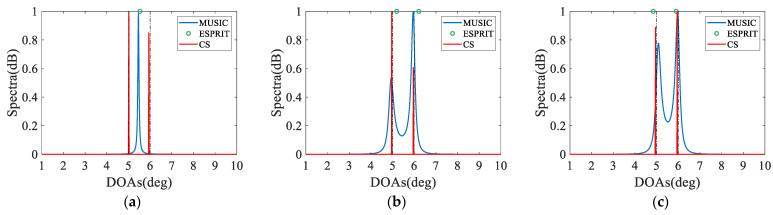

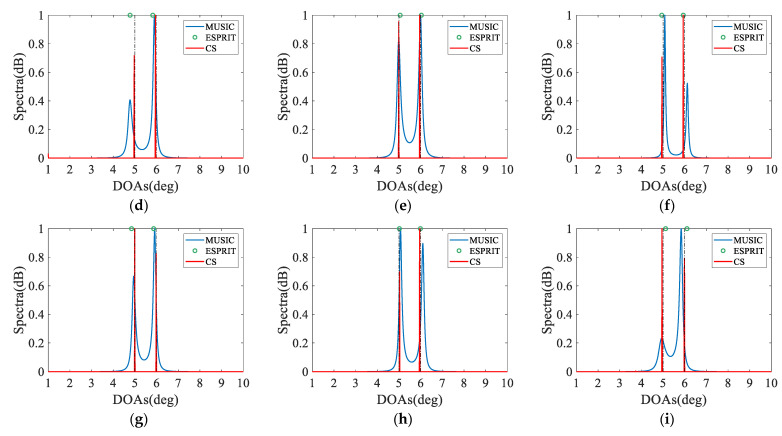
Comparison of resolution, where *N* = 12, *K* = 2, SNR = 0 dB and *L* = 500. The black dashed lines represent the true DOAs. (**a**) CPA; (**b**) NA; (**c**) ENA; (**d**) EoNA; (**e**) NA-TS (*N*_3_ = 1); (**f**) NA-TS (*N*_3_ = 2); (**g**) NA-TS (*N*_3_ = 3); (**h**) NA-TS (*N*_3_ = 4); (**i**) NA-TS (*N*_3_ = 5).

In the third numerical example, we compare the resolution ability of various configurations composed of 12 sensors with different estimators (including MUSIC, ESPRIT and CS). Here, two closely spaced sources impinge from 5° and 6°, whose SNR = 0 dB and the number of snapshots is set to 500. As shown in Figure 5, all configurations can identify both peaks in the true angles with the CS estimators, whereas the estimation results of the subspace-based estimators deviate slightly from the true angles. It is worth noting that CPA fails to resolve the two closely spaced sources with the subspace-based estimators, because which utilize only half of the DOFs, and the reduced virtual aperture severely affects the resolution. It is evident from Figure 5 that the proposed NA-TS (*N*_3_ = 1) realizes a better resolution than other configurations for any estimator due to the enhanced DOFs and virtual aperture.

### 4.4. Root Mean Square Error

In the fourth numerical example, the root mean square error (RMSE) is used to investigate the estimation performance of the proposed NA-TS, which is calculated as

(12) $$\mathrm{RMSE}=\sqrt{\frac{1}{\gamma K}\sum_{i=1}^{\gamma }\sum_{k=1}^{K}{\left({\hat{\theta }}_{k}^{i}-\theta_{k}\right)}^{2}}$$

where γ and K represents the total number of Monte Carlo trials and sources, respectively. ${\hat{\theta }}_{k}^{i}$ represents the estimated DOA of $\theta_{k}$ in the *i*th trial.

**Figure 6:**
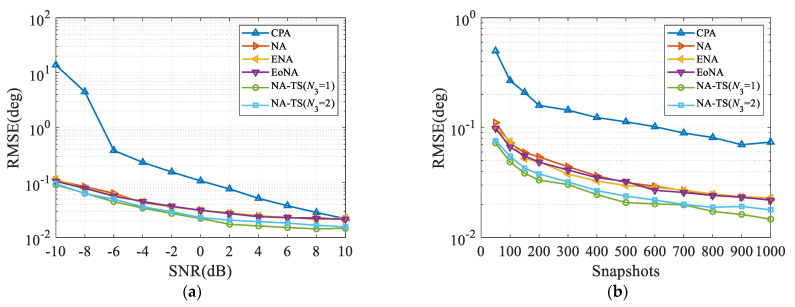
Comparison of RMSE, where two impinging sources are located at 5° and 10°. (**a**) RMSE versus SNR with *L* = 500; (**b**) RMSE versus the number of snapshots with SNR = 0 dB.

Here we use the SS-ESPRIT algorithm [16] to obtain the source to be estimated. It can be observed from Figure 6 that NA-TS obtains a slightly improved performance compared to NA, ENA and EoNA, and a significantly superior estimation performance than CPA due to the increased DOFs. Thus, the proposed NA-TS can acquire better DOA estimation.

### 4.5. Cramér-Rao Lower Bound

**Figure 7:**
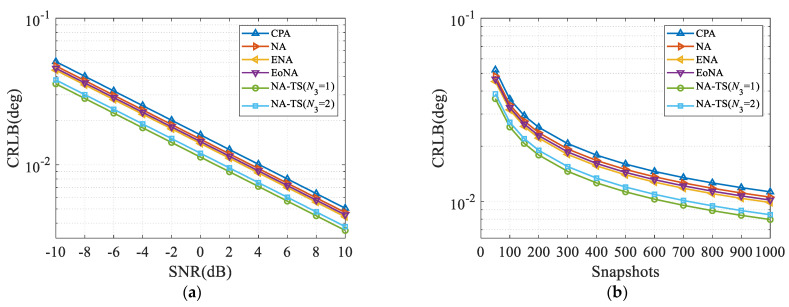
Comparison of CRB, where two sources impinge from 5° and 10°. (**a**) CRB versus SNR with *L* = 500; (**b**) CRB versus the number of snapshots with SNR = 0 dB.

In the last numerical example, the Cramér–Rao Lower Bound (CRLB) [36] performance comparison between various configurations is implemented. It is evident from Figure 7 that NA-TS outperforms CPA, NA, ENA and EoNA due to the increased DOFs and extended array aperture. Consequently, compared to CPA, NA, ENA and EoNA, better estimation performance can be attained by the proposed NA-TS.

## 5. Conclusions

In this paper, we proposed a nested array composed of three sub-ULAs with different interspacing, which has an explicit geometry and a simple closed-form expression for DOFs. Both NA and INA can be regarded as special cases of the proposed NA-TS. In addition, the mathematical derivation proves that NA-TS can generate a hole-free DCA and obtain enhanced DOFs, which further leads to excellent DOA estimation performance. Future work can draw on SNA, ANA and EGNA to mitigate sensor coupling, and investigate the applicability of sensor arrays and estimation algorithms.

## Figures and Tables

**Table 1:** Comparisons of the optimal DOFs and corresponding solutions for relevant arrays.

Arrays	N	$\mathrm{Optimal}\;N_{1},\;N_{2},\;N_{3}$	DOFs
NA	Even	$N_{1}=N/2,\;N_{2}=N/2,\;N_{3}=0$	$N^{2}/2+N-1$
	Odd	$N_{1}=\left(N-1\right)/2,\;N_{2}=\left(N+1\right)/2,\;N_{3}=0$	$N^{2}/2+N-1/2$
EoNA	Even	$N_{1}=N/2,\;N_{2}=N/2,\;N_{3}=0$	$N^{2}/2+N+1$
	Odd	$N_{1}=\left(N-1\right)/2,\;N_{2}=\left(N+1\right)/2,\;N_{3}=0$	$N^{2}/2+N+3/2$
ENA	Even	$N_{1}=N/2,\;N_{2}=N/2,\;N_{3}=0$	$N^{2}/2+N+1$
	Odd	$N_{1}=\left(N-1\right)/2,\;N_{2}=\left(N+1\right)/2,\;N_{3}=0$	$N^{2}/2+N-1/2$
NA-TS	Even	$N_{1}=\left(N-2N_{3}\right)/2,\;N_{2}=N/2,\;1\leq N_{3}<N/2$	$N^{2}/2+2N-2N_{3}-1$
	Odd	$N_{1}=\left(N-2N_{3}-1\right)/2,\;N_{2}=\left(N+1\right)/2,\;1\leq N_{3}<\left(N-1\right)/2$	$N^{2}/2+2N-2N_{3}-3/2$

## Data Availability

The MATLAB codes supporting the results of this research are available from yule_zhang0921@163.com (Y.Z.).

## Appendix A. Proof of Proposition 1

A lag $d\in D^{+}$ is considered according to the difference co-array in Definition 5. We want to show that Proposition 1 holds, which is equivalent to proving that the lag d traverses all integers from 0 to $N_{1}N_{2}+N_{2}N_{3}+N_{1}+N_{2}-1$, i.e.,

(A1) $$0\leq d\leq N_{1}N_{2}+N_{2}N_{3}+N_{1}+N_{2}-1$$

For further analysis, the set $D^{+}$ in Definition 5 is reformulated as

(A2) $$D^{+}=\left\{D_{i,j}\left|D_{i,j}=S_{j}-S_{i},1\leq i\leq j\leq 3\right.\right\}$$

where $D_{i,j}$ represents the difference set obtained by subtracting the elements in the *i*th sub-ULA from the elements in the *j*th sub-ULA.

Then, from (10), we can obtain

(A3) $$D_{1,1}=\underset{\in S_{1}}{\underbrace{\left[s_{1}\right]}}-\underset{\in S_{1}}{\underbrace{\left[s_{1}\right]}}=\left\{0,1,\cdots ,N_{1}-1\right\}$$

where $0\leq s_{1}\leq N_{1}-1$.

(A4) $$D_{2,1}=\underset{\in S_{2}}{\underbrace{\left[N_{1}+\left(N_{1}+1+N_{3}\right)s_{2}\right]}}-\underset{\in S_{1}}{\underbrace{\left[s_{1}\right]}}=\left\{1,2,\cdots ,N_{1}\right\}+\left\{\begin{array}{l} 0\\ \left(N_{1}+1+N_{3}\right)\\ \vdots \\ \left(N_{2}-1\right)\left(N_{1}+1+N_{3}\right) \end{array}\right.$$

where $0\leq s_{1}\leq N_{1}-1$, $0\leq s_{2}\leq N_{2}-1$.

(A5) $$\begin{array}{cc} \; & D_{3,2}=\underset{\in S_{3}}{\underbrace{\left[N_{1}+\left(N_{1}+1+N_{3}\right)\left(N_{2}-1\right)+\left(N_{1}+1\right)+s_{3}\right]}}-\underset{\in S_{2}}{\underbrace{\left[N_{1}+\left(N_{1}+1+N_{3}\right)s_{2}\right]}}\\ \; & =\left(N_{1}+1\right)+\left\{\begin{array}{c} \left\{0,\left(N_{1}+1+N_{3}\right),\cdots ,\left(N_{2}-1\right)\left(N_{1}+1+N_{3}\right)\right\}\\ \left\{0,\left(N_{1}+1+N_{3}\right),\cdots ,\left(N_{2}-1\right)\left(N_{1}+1+N_{3}\right)\right\}+1\\ \vdots \\ \left\{0,\left(N_{1}+1+N_{3}\right),\cdots ,\left(N_{2}-1\right)\left(N_{1}+1+N_{3}\right)\right\}+\left(N_{3}-1\right) \end{array}\right.\\ \; & =\left(N_{1}+1\right)+\left\{\begin{array}{c} \left\{0,1,\cdots ,\left(N_{3}-1\right)\right\}\\ \left\{0,1,\cdots ,\left(N_{3}-1\right)\right\}+\left(N_{1}+1+N_{3}\right)\\ \vdots \\ \left\{0,1,\cdots ,\left(N_{3}-1\right)\right\}+\left(N_{2}-1\right)\left(N_{1}+1+N_{3}\right) \end{array}\right. \end{array}$$

where $0\leq s_{2}\leq N_{2}-1$, $0\leq s_{3}\leq N_{3}-1$.

(A6) $$\begin{array}{c} D_{2,2}=\underset{\in S_{2}}{\underbrace{\left[N_{1}+\left(N_{1}+1+N_{3}\right)s_{2}\right]}}-\underset{\in S_{2}}{\underbrace{\left[N_{1}+\left(N_{1}+1+N_{3}\right)s_{2}\right]}}\\ =\left(N_{1}+1+N_{3}\right)\left\{0,1\cdots ,\left(N_{2}-1\right)\right\} \end{array}$$

where $0\leq s_{2}\leq N_{2}-1$.

(A7) $$\begin{array}{c} D_{3,1}=\underset{\in S_{3}}{\underbrace{\left[N_{1}+\left(N_{1}+1+N_{3}\right)\left(N_{2}-1\right)+\left(N_{1}+1\right)+s_{3}\right]}}-\underset{\in S_{1}}{\underbrace{\left[s_{1}\right]}}\\ =\left(N_{1}+1+N_{3}\right)\left(N_{2}-1\right)+\left(N_{1}+1\right)+\left\{\begin{array}{l} \left\{1,2,\cdots ,N_{1}\right\}\\ \left\{1,2,\cdots ,N_{1}\right\}+1\\ \vdots \\ \left\{1,2,\cdots ,N_{1}\right\}+\left(N_{3}-1\right) \end{array}\right. \end{array}$$

where $0\leq s_{1}\leq N_{1}-1$, $0\leq s_{3}\leq N_{3}-1$.

We can easily find the corresponding elements from (A3)~(A7) to fulfill the lag *d* represented by (A1).

Therefore, in case of $N_{1}\geq 1,\;N_{2}\geq 2,\;N_{3}\geq 1$, the DOFs of the proposed NA-TS is equal to $2\max(d)+1=2\left(N_{1}N_{2}+N_{2}N_{3}+N_{1}+N_{2}\right)-1$. Since $\max(S_{3})$ in (10) and $\max(D_{3,1})$ in (A7) are both equal to $N_{1}N_{2}+N_{2}N_{3}+N_{1}+N_{2}-1$, the NA-TS is a restricted array. This completes the proof.

## Appendix B. Proof of Proposition 2

Based on Proposition 1, the maximal DOFs under the constraint of $N=N_{1}+N_{2}+N_{3}$ can be attributed to the following optimization

(A8) $$\begin{array}{ll} \underset{N_{1},N_{2},N_{3}}{\max} & 2\left(N_{1}N_{2}+N_{2}N_{3}+N_{1}+N_{2}\right)-1\\ s.t. & N=N_{1}+N_{2}+N_{3} \end{array}$$

Fixing $N_{3}$ and using the Arithmetic Mean-Geometric Mean (AM-GM) inequality [10] we can obtain the solutions of (A8) as follows:

(A9) $$\left|D\right|=\left\{\begin{array}{ll} \frac{N^{2}}{2}+2N-2N_{3}-1 & N\;is\;even\;\left(N_{1}=\frac{N-2N_{3}}{2},\;N_{2}=\frac{N}{2},\;1\leq N_{3}<\frac{N}{2}\right)\\ \frac{N^{2}}{2}+2N-2N_{3}-\frac{3}{2} & N\;is\;odd\;\left(N_{1}=\frac{N-2N_{3}-1}{2},\;N_{2}=\frac{N+1}{2},\;1\leq N_{3}<\frac{N-1}{2}\right) \end{array}\right.$$

This completes the proof.
